# Using Waveguides to Model the Dynamic Stiffness of Pre-Compressed Natural Rubber Vibration Isolators

**DOI:** 10.3390/polym13111703

**Published:** 2021-05-23

**Authors:** Michael Coja, Leif Kari

**Affiliations:** The Marcus Wallenberg Laboratory for Sound and Vibration Research (MWL), Department of Engineering Mechanics, KTH Royal Institute of Technology, 100 44 Stockholm, Sweden; michaelcoja@yahoo.fr

**Keywords:** natural rubber vibration isolator, waveguide model, mode matching, pre-compression, wide frequency range, transformation, fractional derivative, Mittag–Leffler function, resonance

## Abstract

A waveguide model for a pre-compressed cylindrical natural rubber vibration isolator is developed within a wide frequency range—20 to 2000 Hz—and for a wide pre-compression domain—from vanishing to the maximum in service, that is 20%. The problems of simultaneously modeling the pre-compression and frequency dependence are solved by applying a transformation of the pre-compressed isolator into a globally equivalent linearized, homogeneous, and isotropic form, thereby reducing the original, mathematically arduous, and complex problem into a vastly simpler assignment while using a straightforward waveguide approach to satisfy the boundary conditions by mode-matching. A fractional standard linear solid is applied as the visco-elastic natural rubber model while using a Mittag–Leffler function as the stress relaxation function. The dynamic stiffness is found to depend strongly on the frequency and pre-compression. The former is resulting in resonance phenomena such as peaks and troughs, while the latter exhibits a low-frequency magnitude stiffness increase in addition to peak and trough shifts with increased pre-compressions. Good agreement with nonlinear finite element results is obtained for the considered frequency and pre-compression range in contrast to the results of standard waveguide approaches.

## 1. Introduction

Natural rubber is an interesting polymeric material. It exhibits frequency- and pre-strain-dependent mechanical properties, including its shear storage and loss modula, and an immense dissimilarity between the transversal and longitudinal wave velocities [1]. The latter is due to its nearly incompressibility and displays similarities to that of water: comparatively soft in shear but hard in compression—in addition to a density close to 1000 kg/m${}^{3}$. Natural rubber has further excellent elastic and mechanical properties including abrasion resistance, tensile strength, and elongation resistance. Natural rubber vibration isolators are frequently used to insulate receiving structures from a source thanks to their efficiency in attenuating structural vibrations. To ensure an optimized utilization, the dynamic stiffness of natural rubber vibration isolators needs to be accurately determined. While steel springs have a well-established mechanical behavior, natural rubber vibration isolators still include features that require more modeling work.

Simplified models such as the classical long rod example are extensively applied to vibration isolators [2,3,4,5], despite some major limitations. The long rod model in which plane cross sections perpendicular to the axial co-ordinate axis are assumed to remain plane and perpendicular to the axial co-ordinate axis and where the stress is assumed to be uniaxial and uniform while radial expansions and contractions are neglected fails to give satisfactory predictions [6]. In particular, the lack of success is frequent for a vastly common vibration isolator—namely, the cylindrical rubber isolator with a large diameter-to-length ratio. The increased influence of higher-order modes, which are necessary to satisfy the boundary conditions, and the increased influence of dispersion explain the lack of correctness of the approximate solution. The dynamic stiffness results of a refined waveguide model [7,8] accounting for those effects are shown to diverge substantially from all the investigated, traditional simple models.

However, the above approaches do not take into account the influence of pre-compression, which is from a practical point of view, an inescapable parameter since most vibration isolators carry the static load of the source or isolated system they are attached to. These effects involve geometrical and material non-linearities, rendering the modeling task substantially more complex. Indeed, the original, free outer surface of the isolator is transformed into a deformed shape, clearly non-identical to the original surface, by the application of the static load—a geometrical non-linearity. The geometrical non-linearity results in a nonlinearity in the axial stiffness due to geometrical changes. In addition, the response to a superimposed dynamic excitation displays a seemingly anisotropic and non-homogeneous feature in the transformed state—a material non-linearity. The material non-linearity results in a nonlinearity in the axial stiffness due to nonlinearity in the stress–strain relation for the material itself. A way to account for the pre-compression dependence is by adopting a nonlinear approach based on single integral viscoelastic constitutive theory. The mechanical response of two natural rubber compounds were examined [9,10] under steady-state torsion oscillations combined with a large static extension while additionally assuming a time–strain separability of the relaxation tensor and displayed good agreement with experiments. Using a similar approach, the dynamic stiffness of a cylindrical vibration isolator was derived [11] for different pre-compressions within an audible frequency range obtaining excellent agreements with a series of experiments. To describe the audible short term response of the rubber, a fractional derivative type relaxation function was successfully adopted [7,11,12,13,14,15,16,17,18,19,20,21,22,23,24,25,26,27,28,29,30,31,32,33,34,35,36,37,38,39,40,41,42,43,44,45,46,47,48,49,50,51,52,53,54,55,56,57,58,59,60,61,62,63,64,65,66,67,68,69,70,71,72,73,74], significantly reducing the number of required material parameters. Visco-elastic models with fractional derivatives have sound bases in polymer molecular theory [75]. The dynamic shear modulus was derived by using the principle of virtual work and by linearizing the constitutive relation at a given prestrain [76]. The model was applied to rubber bushings under simple shear and comparisons to experiments for different pre-compressions displayed good agreement although the frequency range was narrow: 0 to 100Hz [76].

Although good results were obtained using the aforementioned models, the intrinsic non-linearity of the theory of pre-strained isolators, involving a series of complex mathematical difficulties, renders its application arduous. Furthermore, the use of non-linear finite element methods makes the results difficult to interpret as no closed-form solutions are obtained.

The present paper proposes a waveguide approach for the dynamic stiffness modeling of natural rubber vibration isolators accounting for both the pre-compression and frequency dependencies. This original, mathematically cumbersome task is simplified by transforming the complex shape of the pre-compressed natural rubber isolator into a globally equivalent, homogeneous, and isotropic form, combined with a straightforward waveguide approach to satisfy the boundary conditions by mode matching. A fractional standard linear solid is applied while using a Mittag–Leffler function as the stress relaxation function [14]. The proposed method is applied to a cylindrical rubber isolator, where the influence of pre-compression, cylinder length, and diameter are investigated. Comparisons with the results of non-linear finite element method are made.

## 2. Materials and Methods

### 2.1. Vibration Isolator

**Figure 1:**
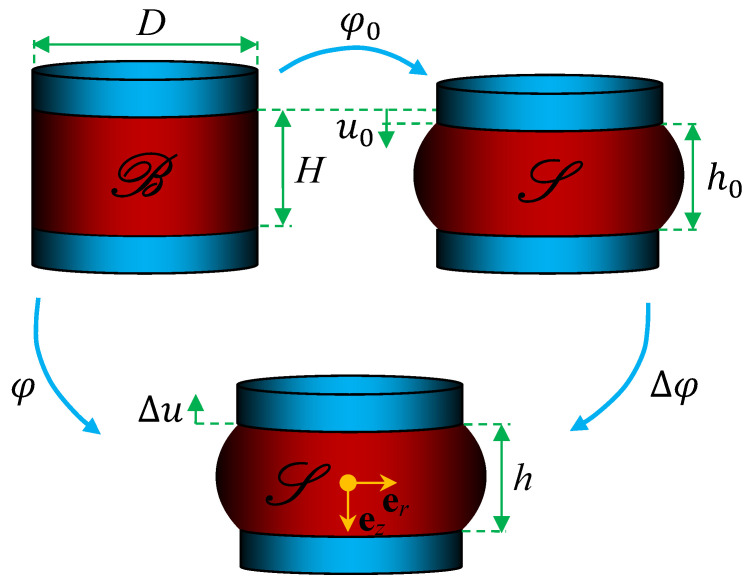
Reference configuration B and current configuration S.

The studied natural rubber isolator in Figure 1 consists of a vulcanized natural rubber cylinder of height *H* and diameter D=2R in its natural state, stress free and undeformed, firmly bonded to two parallel, identical metal plates of mass $m_{\mathrm{mp}}$.

### 2.2. Kinematics

Consider the vibration isolator, a simple body consisting of continuously distributed natural rubber material occupying a reference configuration B in its natural state, and refer to S as an ambient space in which the evolution of the body takes place, where B and S$\subset R^{3}$ are sufficiently smooth, oriented open manifolds. Consider an excitation source applied to the studied natural rubber isolator; the isolator undergoes a regular motion φ:B→S from the reference configuration B to the current S, which is decomposed into $\varphi =\Delta \varphi \circ \varphi_{0}:B\to S\to S$, where the excitation source’s mass load and other applied external static forces result in a finite motion $\varphi_{0}:B\to S$, being the quasi-static natural rubber isolator pre-compression, while a superimposed infinitesimal excitation results in a motion Δφ:S→S. Consequently, the compression displacement boundary condition $u=ue_{z}$ at the upper bonded natural rubber surface in S is decomposable into $u=\left(u_{0}-\Delta u\left(t\right)\right)e_{z}$, where Δu(t) is a superimposed axial displacement, being the infinitesimal dynamic source excitation, and *t* is time.

### 2.3. Equivalent Transformation

**Figure 2:**
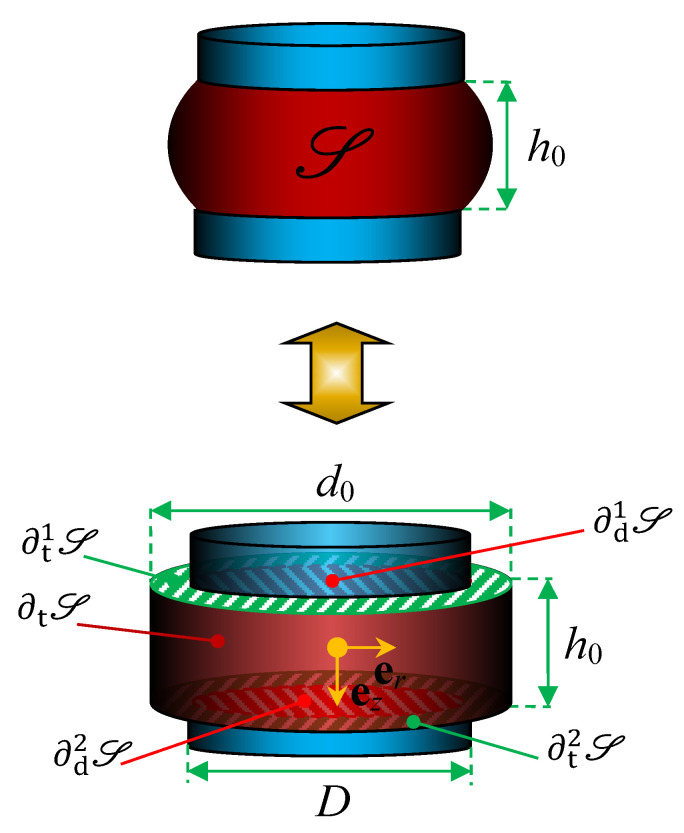
Equivalent transformation in S with boundary surfaces marked.

Accounting for the nearly incompressibility of natural rubber, an approximate globally equivalent configuration is derived from the pre-compressed natural rubber isolator configuration S, as depicted in Figure 2. The unknown deformed shape obtained after the pre-compression is transformed into a straight cylinder of identical volume. Consider the pre-compression $\varphi_{0}$ in Figure 1, resulting in an axial displacement of $u=u_{0}e_{z}$ at the upper bonded natural rubber surface, the equivalent cylinder height and radius in terms of those of the original, natural state cylinder B, read

(1) $$h_{0}\left(u_{0}\right)=H-u_{0}$$

and

(2) $$r_{0}\left(u_{0}\right)=\frac{d_{0}\left(u_{0}\right)}{2}=R\sqrt{\frac{H}{H-u_{0}}},$$

respectively, where $d_{0}$ is diameter.

### 2.4. Quasi-Static Natural Rubber Isolator Pre-Compression

The non-linear relation between the quasi-static axial force $F_{0}$ needed to pre-compress the natural rubber vibration isolator a quasi-static axial displacement of $u_{0}$ [77] reads

(3) $$F_{0}=\frac{3\pi }{4}\frac{\mu_{\infty }D^{2}u_{0}}{H-u_{0}}\left[1+\frac{D^{2}\left(2H-u_{0}\right)}{16H^{2}\left(H-u_{0}\right)}\right]$$

and the corresponding quasi-static stiffness is

(4) $$k_{0}=\frac{dF_{0}}{du_{0}}=\frac{3\pi }{4}\frac{\mu_{\infty }D^{2}H}{{\left(H-u_{0}\right)}^{2}}\left[1+\frac{D^{2}}{8H(H-u_{0})}\right],$$

where $\mu_{\infty }$ is the equilibrium (static) shear modulus. An interested reader is referred to Kari [77] regarding the derivations and validations of the previous quasi-static formulas. The relation (3) is advantageously applied while transforming a known axial pre-load into a quasi-static axial displacement, and vice versa, whilst the relation (4) is successfully applied while finding the quasi-static stiffness at a known axial pre-compression, and vice versa.

### 2.5. General Solution for the Superimposed Motion

The equation of infinitesimal motion in S for an isotropic viscoelastic material in absence of body forces [78,79] reads

(5) $$\rho \frac{\partial^{2}u}{\partial t^{2}}=\int_{-\infty }^{t}\left[\kappa \left(t-\tau \right)+\frac{\mu (t-\tau )}{3}\right]\frac{\partial \nabla \left[\nabla \;\cdot \;u(\tau )\right]}{\partial \tau }d\tau +\int_{-\infty }^{t}\mu \left(t-\tau \right)\frac{\partial \nabla^{2}u\left(\tau \right)}{\partial \tau }d\tau ,$$

where u is the vector displacement field in S; $\nabla^{2}$ is the Laplacian; ρ is the mass per unit volume; and κ and μ are the bulk and shear relaxation function, respectively. A convenient bulk relaxation for natural rubber reads

(6) $$\kappa \left(t\right)=\alpha \mu_{\infty }h\left(t\right),$$

due to the near incompressibility of rubber, where the Heaviside step function is *h*, the nearly incompressibility material parameter is α≫1, and the long-term relaxation function (equilibrium shear modulus) is $\mu_{\infty }=\lim_{t\to \infty }\mu \left(t\right)$. Furthermore, a suitable shear relaxation function reads

(7) $$\mu \left(t\right)=\left[1+▵E_{\beta }\left(-{\left\{\frac{t}{\tau }\right\}}^{\beta }\right)\right]\mu_{\infty }h\left(t\right),$$

while modelling a fractional standard linear solid [14] in shear, where the Mittag–Leffler function is

(8) $$E_{\beta }\left(x\right)=\sum_{n=0}^{\infty }\frac{x^{n}}{\Gamma (1+n\beta )},$$

where the Gamma function $\Gamma \left(z\right)=\int_{0}^{\infty }s^{z-1}e^{-s}ds$, z>0, and where the non-dimensional relaxation density $▵\gg 1:\lim_{t\to 0^{+}}\mu \left(t\right)=\mu_{\infty }\left(1+▵\right)$, the fractional derivative order β:0<β⩽1, and the generalized relaxation time τ⩾0 are material parameters.

The frequency domain solution to the equation can be expressed in terms of scalar and vector potential functions $\tilde{\phi }$ and $\tilde{\psi }$, respectively, where $\left(\tilde{\cdot }\right)=\int_{-\infty }^{\infty }\left(\cdot \right)\exp\left(-i\omega t\right)dt$ denotes temporal Fourier transformation, i is an imaginary unit, and ω is an angular frequency, according to the Helmholtz theorem [79]

(9) $$\tilde{u}=\mathrm{grad}\tilde{\phi }+\mathrm{curl}\tilde{\psi },$$

where $\mathrm{grad}\tilde{\phi }=\nabla \tilde{\phi }$, in which $\tilde{\phi }$ and $\tilde{\psi }$ satisfy the Helmholtz equations:

(10) $$\nabla^{2}\tilde{\phi }+k_{L}^{2}\tilde{\phi }=0$$

and

(11) $$\nabla^{2}\tilde{\psi }+k_{T}^{2}\tilde{\psi }=0,$$

and where $k_{L}$ and $k_{T}$ are the longitudinal and transversal wave number, respectively. They read

(12) $$k_{L}=\omega \sqrt{\frac{3\rho }{3\hat{\kappa }+4\hat{\mu }}}$$

and

(13) $$k_{T}=\omega \sqrt{\frac{\rho }{\hat{\mu }}},$$

where the bulk modulus is

(14) $$\hat{\kappa }=\alpha \mu_{\infty }$$

and the shear modulus is

(15) $$\hat{\mu }=\left(1+\frac{▵{\left(\tau i\omega \right)}^{\beta }}{1+{\left(\tau i\omega \right)}^{\beta }}\right)\mu_{\infty }.$$

In passing, alternative and equivalent forms of the equilibrium shear modulus and the non-dimensional relaxation density are $\mu_{\infty }=\lim_{\omega \to 0}\hat{\mu }\left(\omega \right)$ and $▵\gg 1:\lim_{\omega \to \infty }\hat{\mu }\left(\omega \right)=\mu_{\infty }\left(1+▵\right)$, respectively. The shear modulus (15) is a fractional extension of the shear modulus to the classical standard linear solid, where the viscous element with the stress proportional to the time derivative of the strain is substituted with an element with the stress proportional to the fractional time derivative of order β of the strain. Likewise, the exponential stress relaxation function of the classical standard linear solid is substituted with the Mittag–Leffler stress relaxation function for the fractional standard linear solid. The models coincide as β→1. The interested reader is referred to Koeller [14] for more details.

The displacement field is provided by the Helmholtz theorem (9), reading, in a cylindrical co-ordinate system,

(16) $${\tilde{u}}_{r}=\frac{\partial \tilde{\phi }}{\partial r}+ik_{z}{\tilde{\psi }}_{\varphi },$$

(17) $${\tilde{u}}_{\varphi }=0$$

and

(18) $${\tilde{u}}_{z}=-ik_{z}\tilde{\phi }+\frac{{\tilde{\psi }}_{\varphi }}{r}+\frac{\partial {\tilde{\psi }}_{\varphi }}{\partial r},$$

for the axially symmetric non-torsional case, where the axial dependence is assumed to be $\propto \exp(-ik_{z}z)$ and $k_{z}$ is the axial wave number. The corresponding stress field is

(19) $${\tilde{\sigma }}_{rr}=\left(\alpha \mu_{\infty }-\frac{2\hat{\mu }}{3}\right)\left(\frac{3\alpha \mu_{\infty }+4\hat{\mu }}{3\alpha \mu_{\infty }-2\hat{\mu }}\frac{\partial {\tilde{u}}_{r}}{\partial r}+\frac{{\tilde{u}}_{r}}{r}-ik_{z}{\tilde{u}}_{z}\right),$$

(20) $${\tilde{\sigma }}_{r\varphi }={\tilde{\sigma }}_{\varphi r}=0,$$

(21) $${\tilde{\sigma }}_{rz}={\tilde{\sigma }}_{zr}=\hat{\mu }\left(-ik_{z}{\tilde{u}}_{r}+\frac{\partial {\tilde{u}}_{z}}{\partial r}\right),$$

(22) $${\tilde{\sigma }}_{\varphi \varphi }=\left(\alpha \mu_{\infty }-\frac{2\hat{\mu }}{3}\right)\left(\frac{\partial {\tilde{u}}_{r}}{\partial r}+\frac{3\alpha \mu_{\infty }+4\hat{\mu }}{3\alpha \mu_{\infty }-2\hat{\mu }}\frac{{\tilde{u}}_{r}}{r}-ik_{z}{\tilde{u}}_{z}\right),$$

(23) $${\tilde{\sigma }}_{\varphi z}={\tilde{\sigma }}_{z\varphi }=0$$

and

(24) $${\tilde{\sigma }}_{zz}=\left(\alpha \mu_{\infty }-\frac{2\hat{\mu }}{3}\right)\left(\frac{\partial {\tilde{u}}_{r}}{\partial r}+\frac{{\tilde{u}}_{r}}{r}-ik_{z}\frac{3\alpha \mu_{\infty }+4\hat{\mu }}{3\alpha \mu_{\infty }-2\hat{\mu }}{\tilde{u}}_{z}\right).$$

Using the traction-free boundary condition along the radial surface $\partial_{t}S$ in Figure 2, the dispersion relation for axially symmetric and non-torsional waves in an infinite cylinder [80,81] is given by

(25) $$0=4k_{z}^{2}\left[k_{L}^{2}-k_{z}^{2}\right]\chi \left(R\sqrt{\frac{H(k_{T}^{2}-k_{z}^{2})}{H-u_{0}}}\right)+{\left[k_{T}^{2}-2k_{z}^{2}\right]}^{2}\chi \left(R\sqrt{\frac{H(k_{L}^{2}-k_{z}^{2})}{H-u_{0}}}\right)-2k_{T}^{2}\left[k_{L}^{2}-k_{z}^{2}\right],$$

where the Onoe function $\chi \left(x\right)=xJ_{0}\left(x\right)/J_{1}\left(x\right)$ and $J_{s}$ is the Bessel function of first kind and order *s*.

The dispersion relation solution (25) is calculated by a Newton–Raphson method, with initial values given by a winding integral method, where the search domain is split into branch-cut absent sub-domains [8]. The axial wave number reads $k_{z,n}$, where $n\in Z_{+}$ and labels different solutions, arranged in increasing spatial order, as $|ℑk_{z,n}|\leq |ℑk_{z,m}|;n<m$ and $Z_{+}$ denotes the set of positive integers.

The potential fields solutions to Helmholtz equations (Equations (10) and (11)) can be formulated as follows [7]:

(26) $$\tilde{\phi }=\;\;\sum_{n=1}^{\infty }\left[A_{n}^{+}e^{-ik_{z,n}z}+A_{n}^{-}e^{ik_{z,n}z}\right]J_{0}\left(r\sqrt{k_{L}^{2}-k_{z,n}^{2}}\right),$$

(27) $${\tilde{\psi }}_{\varphi }\;\;=\;\;\sum_{n=1}^{\infty }\;\left[B_{n}^{+}e^{-ik_{z,n}z}+B_{n}^{-}e^{ik_{z,n}z}\right]J_{1}\left(r\sqrt{k_{T}^{2}-k_{z,n}^{2}}\right)$$

and

(28) $${\tilde{\psi }}_{r}={\tilde{\psi }}_{z}=0,$$

where $r\in [0,r_{0}\left(u_{0}\right)]$ and $z\in [-h_{0}\left(u_{0}\right)/2,h_{0}\left(u_{0}\right)/2]$, with the origin of the local system being taken at the center of the natural rubber isolator. The coefficients are interrelated by the traction free boundary condition as $A_{n}^{+}=\Lambda_{n}\left(u_{0}\right)B_{n}^{+}$ and $A_{n}^{-}=-\Lambda_{n}\left(u_{0}\right)B_{n}^{-}$, where

(29) $$\Lambda_{n}\left(u_{0}\right)=\frac{k_{T}^{2}-2k_{z,n}^{2}}{2ik_{z,n}\sqrt{k_{L}^{2}-k_{z,n}^{2}}}\frac{J_{1}\left(R\sqrt{\frac{H(k_{T}^{2}-k_{z,n}^{2})}{H-u_{0}}}\right)}{J_{1}\left(R\sqrt{\frac{H(k_{L}^{2}-k_{z,n}^{2})}{H-u_{0}}}\right)}.$$

The boundary conditions at the cylinder ends are satisfied by mode matching the above fields.

### 2.6. Boundary Conditions

The boundary conditions for the pre-compressed, transformed natural rubber isolator undergoing a superimposed infinitesimal motion Δφ:S→S are vanishing shear and axial stress on $\partial_{t}^{1}S$ and $\partial_{t}^{2}S$ while restraining the radial and axial displacement on $\partial_{d}^{2}S$ and imposing an axial infinitesimal displacement of $-\Delta \tilde{u}e_{z}$ as well as a vanishing radial displacement on $\partial_{d}^{1}S$. The boundary conditions hereby applied to $\bar{\partial_{d}^{1}S\cup \partial_{t}^{1}S\cup \partial_{t}S\cup \partial_{d}^{2}S\cup \partial_{t}^{2}S}=\partial S$ are locally non-mixed and read

(30) $$\tilde{u}\cdot e_{r}=0$$

and

(31) $$\tilde{u}\cdot e_{z}=-\Delta \tilde{u},$$

given on $\partial_{d}^{1}S$;

(32) $$\tilde{u}\cdot e_{r}=0$$

and

(33) $$\tilde{u}\cdot e_{z}=0,$$

given on $\partial_{d}^{2}S$;

(34) $$e_{r}\cdot \left(\tilde{\sigma }\;e_{z}\right)=0,$$

and

(35) $$e_{z}\cdot \left(\tilde{\sigma }\;e_{z}\right)=0,$$

given on $\partial_{t}^{1}S$ and $\partial_{t}^{2}S$ and, finally,

(36) $$\tilde{\sigma }\;e_{r}=0,$$

given on $\partial_{t}S$, with the latter satisfied by the dispersion relation (25). Using (16), (18), (26), (27), and (29), the requirements (30) and (31) become

(37) $$\sum_{n=1}^{\infty }\left(C_{n}^{+}e^{ik_{z,n}\left(H-u_{0}\right)/2}+C_{n}^{-}e^{-ik_{z,n}\left(H-u_{0}\right)/2}\right)V_{n}^{r}\left(r;u_{0}\right)=0$$

and

(38) $$\sum_{n=1}^{\infty }\;\left(C_{n}^{+}e^{ik_{z,n}\left(H-u_{0}\right)/2}-C_{n}^{-}e^{-ik_{z,n}\left(H-u_{0}\right)/2}\right)V_{n}^{z}\left(r;u_{0}\right)=-\Delta \tilde{u},$$

at $z=-h_{0}\left(u_{0}\right)/2$, (32) and (33) become

(39) $$\sum_{n=1}^{\infty }\left(C_{n}^{+}e^{-ik_{z,n}\left(H-u_{0}\right)/2}+C_{n}^{-}e^{ik_{z,n}\left(H-u_{0}\right)/2}\right)V_{n}^{r}\left(r;u_{0}\right)=0$$

and

(40) $$\sum_{n=1}^{\infty }\left(C_{n}^{+}e^{-ik_{z,n}\left(H-u_{0}\right)/2}-C_{n}^{-}e^{ik_{z,n}\left(H-u_{0}\right)/2}\right)V_{n}^{z}\left(r;u_{0}\right)=0,$$

at $z=h_{0}\left(u_{0}\right)/2$, where the coefficients $X_{n}\left(u_{0}\right)C_{n}^{+}=B_{n}^{+}$ and $-X_{n}\left(u_{0}\right)C_{n}^{-}=B_{n}^{-}$ with

(41) $$X_{n}\left(u_{0}\right)=2i\sqrt{k_{L}^{2}-k_{z,n}^{2}}k_{z,n}J_{1}\left(R\sqrt{\frac{H(k_{L}^{2}-k_{z,n}^{2})}{H-u_{0}}}\right)$$

and

(42) $$\begin{array}{c} V_{n}^{r}\left(r;u_{0}\right)=\left(2k_{z,n}^{2}-k_{T}^{2}\right)\sqrt{k_{L}^{2}-k_{z,n}^{2}}J_{1}\left(R\sqrt{\frac{H(k_{T}^{2}-k_{z,n}^{2})}{H-u_{0}}}\right)J_{1}\left(r\sqrt{k_{L}^{2}-k_{z,n}^{2}}\right)\\ \;\;\;\;\;\;\;\;\;\;\;\;\;\;\;\;\;\;\;-2k_{z,n}^{2}\sqrt{k_{L}^{2}-k_{z,n}^{2}}J_{1}\left(R\sqrt{\frac{H(k_{L}^{2}-k_{z,n}^{2})}{H-u_{0}}}\right)J_{1}\left(r\sqrt{k_{T}^{2}-k_{z,n}^{2}}\right) \end{array}$$

and

(43) $$\begin{array}{c} V_{n}^{z}\left(r;u_{0}\right)=ik_{z,n}\left[2k_{z,n}^{2}-k_{T}^{2}\right]J_{1}\left(R\sqrt{\frac{H(k_{T}^{2}-k_{z,n}^{2})}{H-u_{0}}}\right)J_{0}\left(r\sqrt{k_{L}^{2}-k_{z,n}^{2}}\right)\\ \;\;\;\;\;\;\;\;\;\;\;\;\;\;\;\;\;+2ik_{z,n}\sqrt{k_{L}^{2}-k_{z,n}^{2}}\sqrt{k_{T}^{2}-k_{z,n}^{2}}J_{1}\left(R\sqrt{\frac{H(k_{L}^{2}-k_{z,n}^{2})}{H-u_{0}}}\right)J_{0}\left(r\sqrt{k_{T}^{2}-k_{z,n}^{2}}\right), \end{array}$$

r∈[0,R]. Similarly, using (16), (18), (21), (26), (27), and (29), the requirement (34) becomes

(44) $$\sum_{n=1}^{\infty }\left(C_{n}^{+}e^{ik_{z,n}\left(H-u_{0}\right)/2}-C_{n}^{-}e^{-ik_{z,n}\left(H-u_{0}\right)/2}\right)S_{n}^{zr}\left(r;u_{0}\right)=0,$$

at $z=-h_{0}\left(u_{0}\right)/2$, and

(45) $$\sum_{n=1}^{\infty }\left(C_{n}^{+}e^{-ik_{z,n}\left(H-u_{0}\right)/2}-C_{n}^{-}e^{ik_{z,n}\left(H-u_{0}\right)/2}\right)S_{n}^{zr}\left(r;u_{0}\right)=0,$$

at $z=h_{0}\left(u_{0}\right)/2$, where

(46) $$\begin{array}{c} S_{n}^{zr}\left(r;u_{0}\right)=2i\hat{\mu }k_{z,n}\left(k_{T}^{2}-2k_{z,n}^{2}\right)\sqrt{k_{L}^{2}-k_{z,n}^{2}}[J_{1}\left(R\sqrt{\frac{H(k_{T}^{2}-k_{z,n}^{2})}{H-u_{0}}}\right)J_{1}\left(r\sqrt{k_{L}^{2}-k_{z,n}^{2}}\right)\;\;\;\;\;\;\;\;\;\;\\ -J_{1}\left(R\sqrt{\frac{H(k_{L}^{2}-k_{z,n}^{2})}{H-u_{0}}}\right)J_{1}\left(r\sqrt{k_{T}^{2}-k_{z,n}^{2}}\right)], \end{array}$$

$r\in \;]R,r_{0}\left(u_{0}\right)]$. Finally, using (16), (18), (24), (26), (27), and (29), the requirement (35) becomes

(47) $$\sum_{n=1}^{\infty }\left(C_{n}^{+}e^{ik_{z,n}\left(H-u_{0}\right)/2}+C_{n}^{-}e^{-ik_{z,n}\left(H-u_{0}\right)/2}\right)S_{n}^{zz}\left(r;u_{0}\right)=0,$$

at $z=-h_{0}\left(u_{0}\right)/2$, and

(48) $$\sum_{n=1}^{\infty }\left(C_{n}^{+}e^{-ik_{z,n}\left(H-u_{0}\right)/2}+C_{n}^{-}e^{ik_{z,n}\left(H-u_{0}\right)/2}\right)S_{n}^{zz}\left(r;u_{0}\right)=0,$$

at $z=h_{0}\left(u_{0}\right)/2$, where

(49) $$\begin{array}{c} S_{n}^{zz}\left(r;u_{0}\right)=\hat{\mu }\left(k_{T}^{2}-2k_{L}^{2}+2k_{z,n}^{2}\right)\left(2k_{z,n}^{2}-k_{T}^{2}\right)J_{1}\left(R\sqrt{\frac{H(k_{T}^{2}-k_{z,n}^{2})}{H-u_{0}}}\right)J_{0}\left(r\sqrt{k_{L}^{2}-k_{z,n}^{2}}\right)\;\;\;\;\;\;\;\;\;\;\\ +4\hat{\mu }k_{z,n}^{2}\sqrt{k_{T}^{2}-k_{z,n}^{2}}\sqrt{k_{L}^{2}-k_{z,n}^{2}}J_{1}\left(R\sqrt{\frac{H(k_{L}^{2}-k_{z,n}^{2})}{H-u_{0}}}\right)J_{0}\left(r\sqrt{k_{T}^{2}-k_{z,n}^{2}}\right), \end{array}$$

$r\in \;]R,r_{0}\left(u_{0}\right)]$.

The simplest way is to determine the unknown coefficients in (37) to (40), (44), (45), (47), and (48) is to apply a point-matching technique, but in most cases, more accurate results are obtained using the subregion method, dividing the domain into sub-domains. The cylinder ends may be divided into $P_{d}+P_{t}\in Z_{+}$ axially symmetric circular bands between the radii $0=a_{0}<a_{1}<a_{2}<\cdots <a_{P_{d}}=R<a_{P_{d}+1}<a_{P_{d}+2}<\cdots <a_{P_{d}+P_{t}}=r_{0}\left(u_{0}\right)$. By performing integrations between subsequent radii and using the relations $\int xJ_{0}\left(x\right)dx=xJ_{1}\left(x\right)$ and $\int xJ_{1}\left(x\right)dx=\pi x\left(\Xi_{0}\left(x\right)J_{1}\left(x\right)-\Xi_{1}\left(x\right)J_{0}\left(x\right)\right)/2$, where $\Xi_{j}$ is a Struve function of order *j* [82], the conditions (37)–(40) read

(50) $$\sum_{n=1}^{N}\left[D_{n}^{+}e^{ik_{z,n}\left(H-u_{0}\right)/2}+D_{n}^{-}e^{-ik_{z,n}\left(H-u_{0}\right)/2}\right]\left[U_{n}^{r}\left(a_{p};u_{0}\right)-U_{n}^{r}\left(a_{p-1};u_{0}\right)\right]=0,$$

(51) $$\sum_{n=1}^{N}\left[D_{n}^{+}e^{ik_{z,n}\left(H-u_{0}\right)/2}-D_{n}^{-}e^{-ik_{z,n}\left(H-u_{0}\right)/2}\right]\left[U_{n}^{z}\left(a_{p};u_{0}\right)-U_{n}^{z}\left(a_{p-1};u_{0}\right)\right]=\pi \left[a_{p-1}^{2}-a_{p}^{2}\right],$$

(52) $$\sum_{n=1}^{N}\left[D_{n}^{+}e^{-ik_{z,n}\left(H-u_{0}\right)/2}+D_{n}^{-}e^{ik_{z,n}\left(H-u_{0}\right)/2}\right]\left[U_{n}^{r}\left(a_{p};u_{0}\right)-U_{n}^{r}\left(a_{p-1};u_{0}\right)\right]=0$$

and

(53) $$\sum_{n=1}^{N}\left[D_{n}^{+}e^{-ik_{z,n}\left(H-u_{0}\right)/2}-D_{n}^{-}e^{ik_{z,n}\left(H-u_{0}\right)/2}\right]\left[U_{n}^{z}\left(a_{p};u_{0}\right)-U_{n}^{z}\left(a_{p-1};u_{0}\right)\right]=0,$$

where

(54) $$\begin{array}{c} U_{n}^{r}\left(r;u_{0}\right)=\pi^{2}r[\left(k_{T}^{2}-2k_{z,n}^{2}\right)J_{1}\left(R\sqrt{\frac{H(k_{T}^{2}-k_{z,n}^{2})}{H-u_{0}}}\right)W\left(r\sqrt{k_{L}^{2}-k_{z,n}^{2}}\right)\;\;\;\;\;\;\;\;\;\;\;\;\;\;\;\;\\ +2k_{z,n}^{2}\sqrt{\frac{k_{L}^{2}-k_{z,n}^{2}}{k_{T}^{2}-k_{z,n}^{2}}}J_{1}\left(R\sqrt{\frac{H(k_{L}^{2}-k_{z,n}^{2})}{H-u_{0}}}\right)W\left(r\sqrt{k_{T}^{2}-k_{z,n}^{2}}\right)], \end{array}$$

(55) $$\begin{array}{c} U_{n}^{z}\left(r;u_{0}\right)=2i\pi k_{z,n}r[\frac{\left(2k_{z,n}^{2}-k_{T}^{2}\right)}{\sqrt{k_{L}^{2}-k_{z,n}^{2}}}J_{1}\left(R\sqrt{\frac{H(k_{T}^{2}-k_{z,n}^{2})}{H-u_{0}}}\right)J_{1}\left(r\sqrt{k_{L}^{2}-k_{z,n}^{2}}\right)\;\;\;\;\;\;\;\;\;\;\;\;\;\;\;\\ +2\sqrt{k_{L}^{2}-k_{z,n}^{2}}\;J_{1}\left(R\sqrt{\frac{H(k_{L}^{2}-k_{z,n}^{2})}{H-u_{0}}}\right)J_{1}\left(r\sqrt{k_{T}^{2}-k_{z,n}^{2}}\right)], \end{array}$$

$W=J_{0}\Xi_{1}-J_{1}\Xi_{0}$, $D_{n}^{+}\Delta \tilde{u}=C_{n}^{+},D_{n}^{-}\Delta \tilde{u}=C_{n}^{-}$ and $p=1,2,\ldots ,P_{d}$ with $\Delta \tilde{u}\neq 0$. Similarly, the conditions (44), (45), (47), and (48) read

(56) $$\sum_{n=1}^{N}\left[D_{n}^{+}e^{ik_{z,n}\left(H-u_{0}\right)/2}-D_{n}^{-}e^{-ik_{z,n}\left(H-u_{0}\right)/2}\right]\left[T_{n}^{zr}\left(a_{q};u_{0}\right)-T_{n}^{zr}\left(a_{q-1};u_{0}\right)\right]=0,$$

(57) $$\sum_{n=1}^{N}\left[D_{n}^{+}e^{-ik_{z,n}\left(H-u_{0}\right)/2}-D_{n}^{-}e^{ik_{z,n}\left(H-u_{0}\right)/2}\right]\left[T_{n}^{zr}\left(a_{q};u_{0}\right)-T_{n}^{zr}\left(a_{q-1};u_{0}\right)\right]=0,$$

(58) $$\sum_{n=1}^{N}\left[D_{n}^{+}e^{ik_{z,n}\left(H-u_{0}\right)/2}+D_{n}^{-}e^{-ik_{z,n}\left(H-u_{0}\right)/2}\right]\left[T_{n}^{zz}\left(a_{q};u_{0}\right)-T_{n}^{zz}\left(a_{q-1};u_{0}\right)\right]=0,$$

and

(59) $$\sum_{n=1}^{N}\left[D_{n}^{+}e^{-ik_{z,n}\left(H-u_{0}\right)/2}+D_{n}^{-}e^{ik_{z,n}\left(H-u_{0}\right)/2}\right]\left[T_{n}^{zz}\left(a_{q};u_{0}\right)-T_{n}^{zz}\left(a_{q-1};u_{0}\right)\right]=0,$$

where

(60) $$\begin{array}{c} T_{n}^{zr}\left(r;u_{0}\right)=2\pi^{2}ik_{z,n}r\hat{\mu }\left(k_{T}^{2}-2k_{z,n}^{2}\right)\sqrt{k_{L}^{2}-k_{z,n}^{2}}[J_{1}\left(R\sqrt{\frac{H(k_{L}^{2}-k_{z,n}^{2})}{H-u_{0}}}\right)\frac{W(r\sqrt{k_{T}^{2}-k_{z,n}^{2}})}{\sqrt{k_{T}^{2}-k_{z,n}^{2}}}\;\;\;\;\;\;\;\;\;\;\\ -J_{1}\left(R\sqrt{\frac{H(k_{T}^{2}-k_{z,n}^{2})}{H-u_{0}}}\right)\frac{W(r\sqrt{k_{L}^{2}-k_{z,n}^{2}})}{\sqrt{k_{L}^{2}-k_{z,n}^{2}}}], \end{array}$$

(61) $$\begin{array}{c} T_{n}^{zz}\left(r;u_{0}\right)=2\pi r\hat{\mu }[\left(k_{T}^{2}-2k_{L}^{2}+2k_{z,n}^{2}\right)\left(2k_{z,n}^{2}-k_{T}^{2}\right)J_{1}\left(R\sqrt{\frac{H(k_{T}^{2}-k_{z,n}^{2})}{H-u_{0}}}\right)\frac{J_{1}\left(r\sqrt{k_{L}^{2}-k_{z,n}^{2}}\right)}{\sqrt{k_{L}^{2}-k_{z,n}^{2}}}\;\;\;\;\;\;\;\;\;\\ +4k_{z,n}^{2}\sqrt{k_{L}^{2}-k_{z,n}^{2}}J_{1}\left(R\sqrt{\frac{H(k_{L}^{2}-k_{z,n}^{2})}{H-u_{0}}}\right)J_{1}\left(r\sqrt{k_{T}^{2}-k_{z,n}^{2}}\right)] \end{array}$$

and $q=P_{d}+1,P_{d}+2,\ldots ,P_{d}+P_{t}$. To obtain a finite number of equations, the series (50) to (53) and (56) to (59) are truncated after $N\in Z_{+}$ terms, where $P_{d}+P_{t}\geq N/2$.

The relations (50) to (53) and (56) to (59) may be written in a matrix form as Ax=b, where A is a $4(P_{d}+P_{t})\times 2N$ system matrix, x is an unknown coefficient vector ${\left(D_{1}^{+},D_{1}^{-},D_{2}^{+},D_{2}^{-},\ldots ,D_{N}^{+},D_{N}^{-}\right)}^{T}$, and b is a known right-hand vector

(62) $$b=\pi {\left(\underset{P_{d}}{\underset{︸}{0,0,\ldots ,0}},\underset{P_{d}}{\underset{︸}{a_{0}^{2}-a_{1}^{2},a_{1}^{2}-a_{2}^{2},\ldots ,a_{P_{d}-1}^{2}-a_{P_{d}}^{2}}},\underset{2P_{d}+4P_{t}}{\underset{︸}{0,0,\ldots ,0}}\right)}^{T},$$

where T is a transpose. The equation system is solved by singular-value decomposition after rescaling and equilibration [7].

Finally, the transfer and driving point stiffness read

(63) $${\tilde{k}}_{t}\left(\omega ;u_{0}\right)=\frac{1}{\Delta \tilde{u}}\int_{\partial_{d}^{2}S}{\tilde{\sigma }}_{zz}\left(\omega ;u_{0}\right)d\;\left(\partial S\right)$$

and

(64) $${\tilde{k}}_{\mathrm{dp}}\left(\omega ;u_{0}\right)=\frac{1}{\Delta \tilde{u}}\int_{\partial_{d}^{1}S}{\tilde{\sigma }}_{zz}\left(\omega ;u_{0}\right)d\;\left(\partial S\right)-m_{\mathrm{mp}}\omega^{2},$$

respectively, where frequency and pre-compression dependencies are explicitly stated with an assumed rigid body motion of the metal mass $m_{\mathrm{mp}}$. Consequently, they read

(65) $${\tilde{k}}_{t}\left(\omega ;u_{0}\right)=\sum_{n=1}^{N}T_{n}\left(\omega ;u_{0}\right)\left[D_{n}^{+}e^{-ik_{z,n}\left(H-u_{0}\right)/2}+D_{n}^{-}e^{ik_{z,n}\left(H-u_{0}\right)/2}\right]$$

and

(66) $${\tilde{k}}_{\mathrm{dp}}\left(\omega ;u_{0}\right)=\sum_{n=1}^{N}T_{n}\left(\omega ;u_{0}\right)\left[D_{n}^{+}e^{ik_{z,n}\left(H-u_{0}\right)/2}+D_{n}^{-}e^{-ik_{z,n}\left(H-u_{0}\right)/2}\right]-m_{\mathrm{mp}}\omega^{2},$$

where $k_{z,n}=k_{z,n}\left(\omega ;u_{0}\right)$, $D_{n}^{+}=D_{n}^{+}\left(\omega ;u_{0}\right)$, $D_{n}^{-}=D_{n}^{-}\left(\omega ;u_{0}\right)$ and $T_{n}\left(\omega ;u_{0}\right)\equiv T_{n}^{zz}\left(R;u_{0}\right)$, with the latter given by expression (61) with r=R. The series (65) and (66) model the dynamic stiffness to any desired accuracy for a sufficient number of modes *N* while satisfying the traction free boundary condition at the radial surface exactly and the displacement and traction boundary conditions at the cylinder ends in mean. They globally represent the dynamic stiffness for a pre-compressed natural rubber vibration isolator as given by a waveguide model, thereby reducing an original, mathematically arduous, and complex non-linear problem into a vastly simpler assignment.

## 3. Results and Discussion

The formulas above are implemented in Lahey Fortran 90^®^ (Lahey Computer Systems Inc., Incline Village, NV, USA) with all calculations performed in double precision and the results presented using Matlab^®^ (MathWorks Inc., Natick, MA, USA). Two test objects are studied with a diameter of D=100 mm and heights of H=25 and 50 mm, respectively. The two test objects selected represent a normal range of shape factors S=D/4L [1] for this vastly common vibration isolator—with S spanning from 0.5 to 1.

### 3.1. Natural Rubber Material

The natural rubber material applied in this numerical investigation is supposed to be identical to that of Kari et al. [20]—namely, unfilled sulphur cured natural rubber General Purpose Standard Malaysian Rubber (GP SMR) with the ingredients given in Table 1. The fitted material parameters are given in Table 2. The interested reader is referred to Kari [20] for more details regarding the material manufacturing process, the measurement methods and equipments, and the material parameter fitting methods.

### 3.2. Model Results

To obtain accurate stiffness results, the number of modes is required to be large; N=128 is sufficient, while the equation system is taken to be threefold over-determined; $P=P_{d}+P_{t}=256$, with subregion applied radii

(67) $$a_{\xi }\left(u_{0}\right)=\frac{\xi R}{P}\sqrt{\frac{H}{H-u_{0}}},$$

for all integers ξ∈[0,P] obeying $\xi \neq P_{d}$, together with radius $a_{P_{d}}=R$, where the specific $P_{d}\left(u_{0}\right)\in \left[0,P\right]$ satisfies

(68) $$\min_{P_{d}}\left|\frac{P_{d}}{P}\sqrt{\frac{H}{H-u_{0}}}-1\right|.$$

The expression (67) slices the radius from 0 to $r_{0}$ (see Equation (2)) into (P−2) equally distant segments. The remaining two segments between the points $\left(P_{d}-1\right)$ to $P_{d}$ and $P_{d}$ to $\left(P_{d}+1\right)$ are forced to coincide with *R* at the point $P_{d}$. The latter is performed by setting $a_{P_{d}}=R$, with $P_{d}$ given by the expression (68).

For all frequencies considered, 20 to 2000Hz with a step of 10Hz, the effective rank of the conditionalized system matrix is full, that is, 256. The frequency range selected covers two important frequency decades within the audible frequency range.

**Figure 3:**
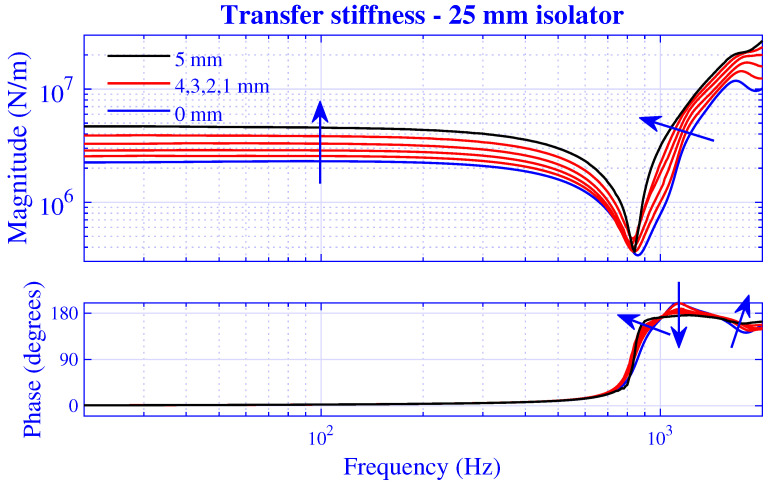
Transfer stiffness magnitude and phase versus frequency for H=25 mm natural rubber isolator at pre-compressions $u_{0}=0,1,2,3,4,$ and 5 mm. Direction for increased compression is marked.

**Figure 4:**
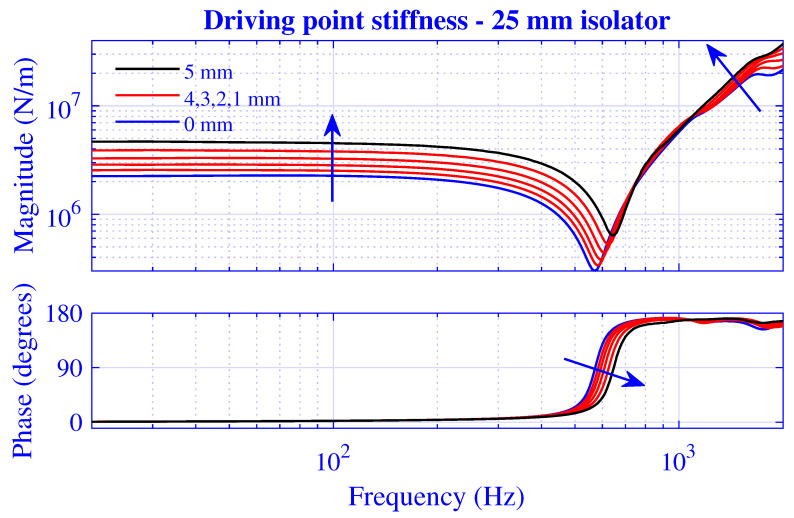
Driving point stiffness magnitude and phase versus frequency for H=25 mm natural rubber isolator at pre-compressions $u_{0}=0,1,2,3,4,$ and 5 mm. Direction for increased compression is marked.

**Figure 5:**
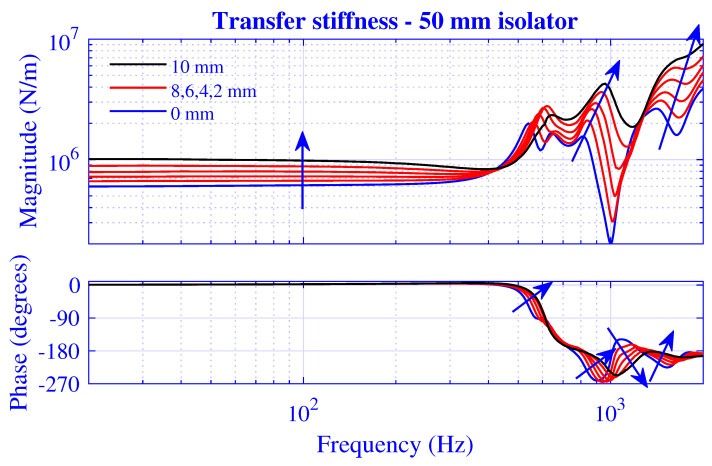
Transfer stiffness magnitude and phase versus frequency for H=50 mm natural rubber isolator at pre-compressions $u_{0}=0,2,4,6,8,$ and 10 mm. Direction for increased compression is marked.

**Figure 6:**
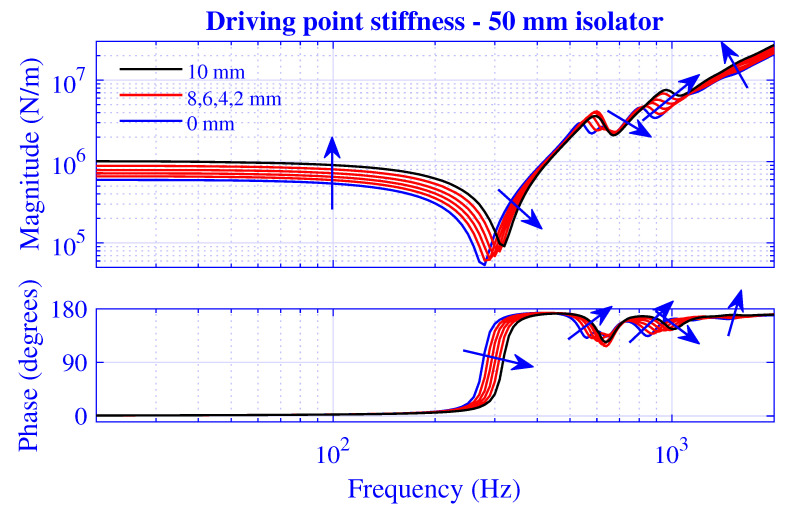
Driving point stiffness magnitude and phase versus frequency for H=50 mm natural rubber isolator at pre-compressions $u_{0}=0,2,4,6,8,$ and 10 mm. Direction for increased compression is marked.

The stiffness magnitude $|\tilde{k}|$ and phase $180∠\tilde{k}/\pi$ for H=25 mm natural rubber isolator at pre-compressions $u_{0}=0,1,2,3,4$ and 5 mm—that is, from vanishing to 20% compression—are presented in Figure 3 and Figure 4, where $\tilde{k}=\left|\tilde{k}\right|\exp\left(i∠\tilde{k}\right)$. Figure 3 displays the transfer stiffness, while the driving point stiffness is in Figure 4. The direction for increased pre-compression is marked by arrows. The corresponding transfer and driving point stiffness results for H=50 mm natural rubber isolator are presented in Figure 5 and Figure 6 at pre-compressions $u_{0}=0,2,4,6,8$, and 10 mm. The driving point stiffness is presented here and in the following without the plate mass contribution. To include this contribution, ${\tilde{k}}_{\mathrm{dp}}{|}_{\mathrm{no}\;\mathrm{mass}}$ is simply replaced by ${\tilde{k}}_{\mathrm{dp}}{{|}_{\mathrm{mass}}={\tilde{k}}_{\mathrm{dp}}|}_{\mathrm{no}\;\mathrm{mass}}-m_{\mathrm{mp}}\omega^{2}$, as in Equation (66). The transfer stiffness is however independent of that mass.

Clearly, the low-frequency stiffness magnitude in Figure 3 is plateau-like at the vanishing pre-compression. Then, the curve drops steeply into a trough at 860Hz and rises sharply while displaying minor oscillations. With respect to the magnitude curve and to the behavior of the phase curve, the trough most likely corresponds to a resonance. At a resonance for elastic materials (that is, with no material damping), the stiffness shows a magnitude trough and a phase jump of $+180^{\circ }$, while displaying a magnitude peak and a phase jump of $-180^{\circ }$ at an anti-resonance. Introducing damping, the resonances and anti-resonances are blunted; in general, the magnitude troughs and peaks are rounded, while the sudden phase jumps disappear, showing a ‘slower’ phase shift. Therefore, it may become difficult to distinguish individual resonances and anti-resonances, particularly for high damping material (such as rubber) at closely spaced resonances and anti-resonances. In passing, it may be observed that it is *not possible* to predict a transfer stiffness resonance by traditional, simplified models—such as the classical long rod example [2,3,4,5]—in contrast to the waveguide approach applied in this paper.

Regarding pre-compression dependence in Figure 3, the low-frequency magnitude plateau shows an increase in compression, as expected. This magnitude increase is also evident in the high-frequency region. However, the trough frequency is almost pre-compression independent, only showing an initial, slight frequency decrease with compression.

The driving point stiffness result in Figure 4 displays a similar behavior as its transfer counterpart. However, the stiffness trough frequency is lower, 570Hz at the vanishing pre-compression, while displaying a clear frequency increase with compression.

The stiffness results for H=50 mm natural rubber isolator in Figure 5 and Figure 6 are similar to those of the shorter natural rubber isolator: displaying a low-frequency stiffness magnitude plateau that increases with pre-compression. However, the transfer stiffness magnitude in Figure 5 rises steeply to peak at the right end of the plateau region while displaying a strong frequency dependence—resulting in peaks and troughs—after this initial anti-resonance. On the other hand, the driving point stiffness magnitude in Figure 6 drops into a deep trough after the low-frequency plateau region—in line with the shorter natural rubber isolator—while rising steeply after this initial resonance, though eventually showing a stronger high-frequency oscillation compared to that of the shorter natural rubber isolator within the considered frequency range. The driving point stiffness resonance and anti-resonance succeed each other repeatedly in Figure 4 and Figure 6, as $0\leq ∠{\tilde{k}}_{\mathrm{dp}}\leq \pi$ from thermodynamically point of view. However, this alternating order is not necessary for the transfer stiffness in Figure 3 and Figure 5 as no such phase restriction exists for that stiffness.

### 3.3. Finite Element Comparison

The developed waveguide model for a pre-compressed natural rubber isolator is verified by calculating the dynamic stiffness of the original, pre-compressed natural rubber isolator in Figure 1 using a non-linear finite element method [11]. To this end, a hyperelastic neo-Hookean material model with a fractional standard linear solid is adopted for the studied, unfilled rubber, where parameters are fitted to match those of the waveguide model under infinitesimal strains, that is, resulting in identical shear modulus, bulk modulus, and density. The mesh employed consists of 3000(50×60) axially symmetric hybrid elements, gradually refined towards the rubber cylinder corners. The force needed for the stiffness calculation is evaluated by spatial integration of extrapolated Gauss point stresses at massless, almost rigid, elastic endplates bonded to the rubber cylinder, thereby avoiding problems at the rubber corners while pre-compressed, resulting in enhanced force estimations. Furthermore, the Lagrangian 9-node (biquadratic displacement–bilinear pressure) and the 4-node (bilinear displacement–constant pressure) hybrid elements, result in infinitesimal stiffness differences, thereby in practice verifying the non-linear finite element solution convergence. The interested reader is referred to Kari [11] for more details concerning the implementation and validation of the non-linear finite element method.

**Figure 7:**
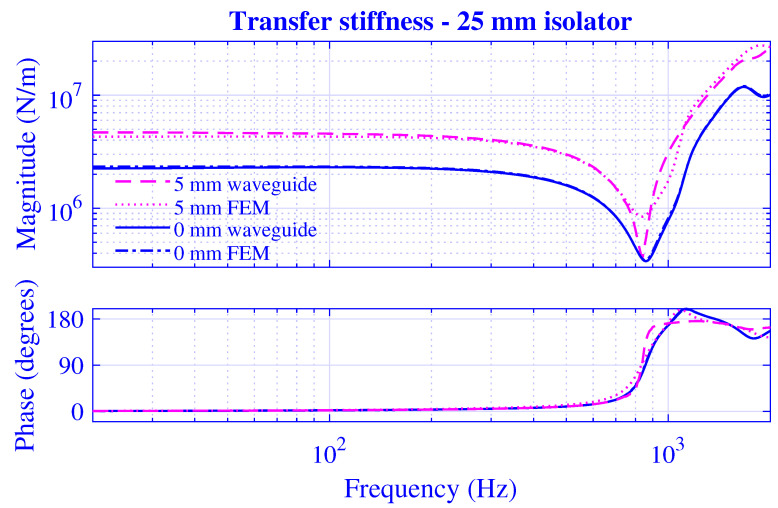
Transfer stiffness magnitude and phase versus frequency for H=25 mm natural rubber isolator at vanishing and maximum pre-compressions. Effective waveguide and non-linear finite element solution.

**Figure 8:**
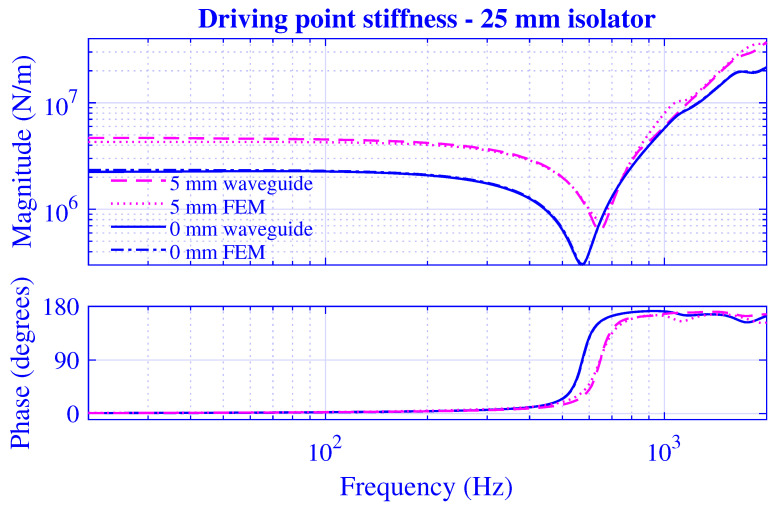
Driving point stiffness magnitude and phase versus frequency for H=25 mm natural rubber isolator at vanishing and maximum pre-compressions. Effective waveguide and non-linear finite element solution.

**Figure 9:**
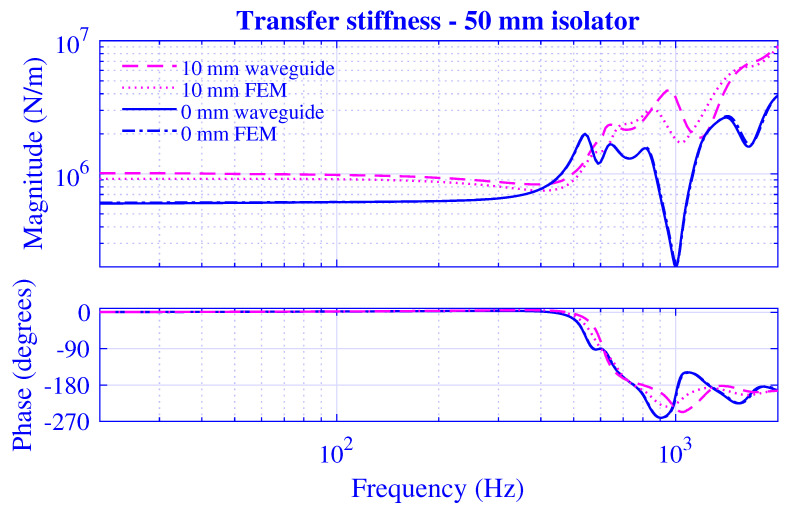
Transfer stiffness magnitude and phase versus frequency for H=50 mm natural rubber isolator at vanishing and maximum pre-compressions. Effective waveguide and non-linear finite element solution.

**Figure 10:**
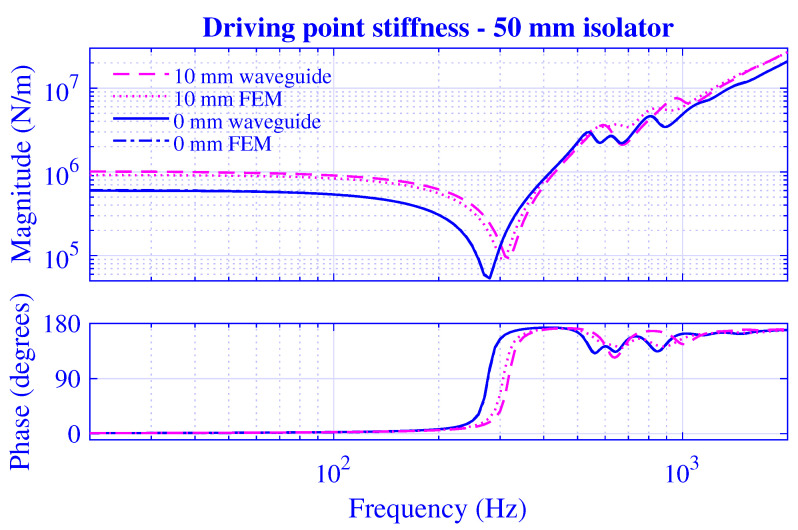
Driving point stiffness magnitude and phase versus frequency for H=50 mm natural rubber isolator at vanishing and maximum pre-compressions. Effective waveguide and non-linear finite element solution.

The transfer and driving point stiffness magnitude and phase for H=25 mm natural rubber isolator at vanishing and maximum pre-compressions; that is, $u_{0}=0$ and 5 mm, are presented in Figure 7 and Figure 8. The corresponding stiffness results for H=50 mm natural rubber isolator are presented in Figure 9 and Figure 10 at pre-compressions $u_{0}=0$ and 10 mm.

Obviously, the waveguide model results closely follow the non-linear finite element solutions in Figure 7, Figure 8, Figure 9 and Figure 10. The stiffness magnitude and phase curves are in practice overlapping at the vanishing pre-compression throughout the whole frequency range. Furthermore, the waveguide stiffness magnitude curves display a slight low-frequency over-estimation while both waveguide stiffness magnitude and phase curves display comparatively close high-frequency agreements with the corresponding non-linear finite element results at the maximum pre-compression. The developed waveguide model presented in this paper is thereby in practice verified. Furthermore, it may be noted that the close agreement between the waveguide model results and those of the non-linear finite element model at the maximum pre-compressions implies that the non-linear geometrical effects prevails over the non-linear material effects in this study since the waveguide model presumes linear material as opposed to the finite element model. This is not surprisingly as the non-linear material model applied is hyperelastic neo-Hookean, being the straightforward non-linear extension of the linear elastic Hookean model and a suitable model for the unfilled natural rubber material virtually employed.

### 3.4. Convergence Properties

The stiffness convergence of the presented waveguide model is investigated by extending the number of radii $P=P_{d}+P_{t}$ and modes *N*.

**Figure 11:**
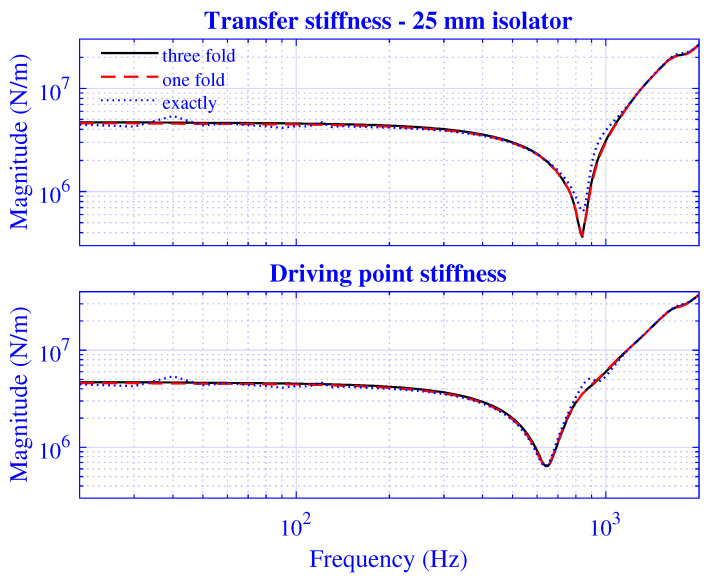
Transfer and driving point stiffness magnitude versus frequency for H=25 mm natural rubber isolator at maximum pre-compression $u_{0}=5\;$ mm. Exactly determined (P=64), single-fold (P=128) and threefold (P=256) over-determined equation system with a number of modes N=128.

**Figure 12:**
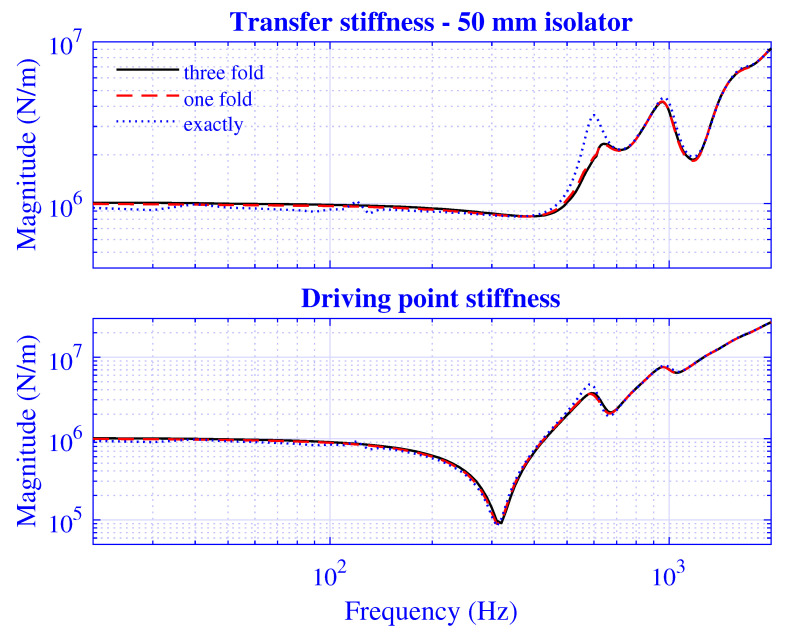
Transfer and driving point stiffness magnitude versus frequency for H=50 mm natural rubber isolator at maximum pre-compression $u_{0}=5\;$ mm. Exactly determined (P=64), and single-fold (P=128) and threefold (P=256) over-determined equation systems with a number of modes N=128.

The *P*-extension is processed by calculating the stiffness for $P=2^{\gamma }$, where γ=6,7,8, while using N=128; that is, the equation system is exactly determined, single, and threefold over-determined, respectively. The transfer and driving point stiffness magnitude for H=25 mm natural rubber isolator at maximum pre-compression $u_{0}=5\;$ mm are presented in Figure 11. The corresponding stiffness results for H=50 mm natural rubber isolator are presented in Figure 12 at maximum pre-compression $u_{0}=10\;$ mm.

Clearly, the P=64 stiffness curves in Figure 11 and Figure 12 oscillate slightly around the well-behaved, almost coinciding P=128 and 256 curves. In practice, it is therefore sufficient to apply a single-fold or threefold over-determined equation system for the stiffness calculation of the object under study focus, while an exactly determined equation system results in an insufficient stiffness solution.

**Figure 13:**
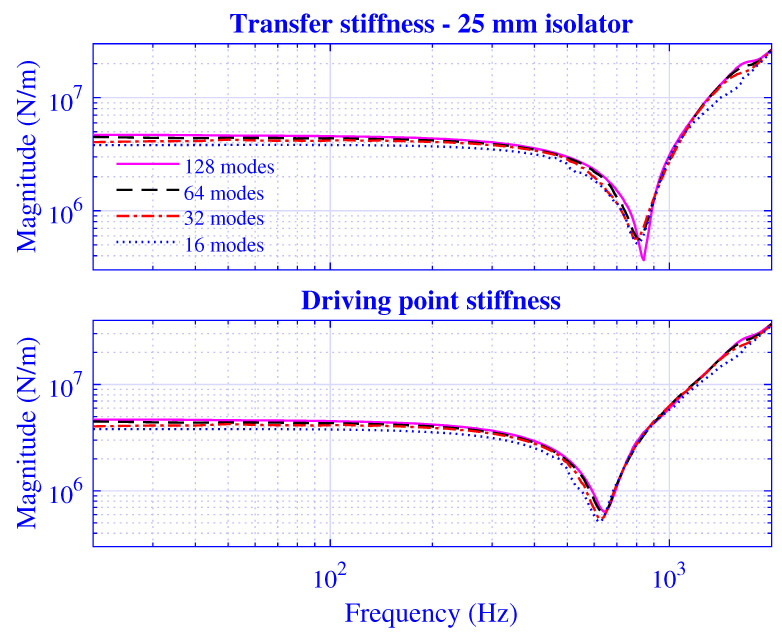
Transfer and driving point stiffness magnitude versus frequency for H=25 mm natural rubber isolator at maximum pre-compression $u_{0}=10\;$ mm. Number of modes N=16,32,64, and 128, using a threefold over-determined equation system (P=2N).

**Figure 14:**
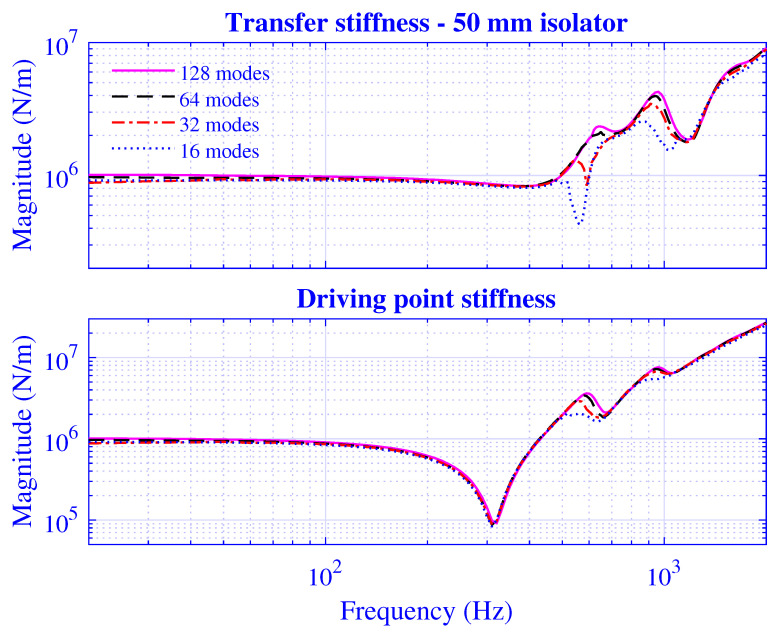
Transfer and driving point stiffness magnitude versus frequency for H=50 mm natural rubber isolator at maximum pre-compression $u_{0}=10\;$ mm. Number of modes N=16,32,64, and 128, using a threefold over-determined equation system (P=2N).

The *N*-extension is processed by calculating the stiffness for $N=2^{\varepsilon }$, where ε=4,5,6,7, while using a threefold over-determined equation system; that is, the number of radii is concurrently extended according to P=2N. The transfer and driving point stiffness magnitude for H=25 mm natural rubber isolator at maximum pre-compression $u_{0}=5\;$ mm are presented in Figure 13, while the corresponding results for H=50 mm natural rubber isolator are presented in Figure 14 at maximum pre-compression $u_{0}=10\;$ mm.

Clearly, the stiffness converges for larger *N*, and the difference between the N=64 and 128 solutions is negligible. In passing, it should be noted that variations in manufacturing process parameters such as curing time, temperature, and pressure; the unbridled compound ingredients; and mixing result in stiffness variations of the individual natural rubber vibration isolators, thus, in practice, limiting any exact agreements between the modelling and measurement results of the individual samples [83]. Furthermore, although the stiffness solution is shown to converge for larger *N*, the necessary number of modes is rather high: N≥64. Physically, these high-order modes contribute to the fulfillment of the displacement and stress boundary conditions at the cylinder ends. The stress field at these ends in turn determines the force needed for the dynamic stiffness calculation, with the latter being the global variable for which the convergence properties are investigated. In practice, the stiffness solution convergence of the developed waveguide model is thereby verified.

### 3.5. Edge Boundary Condition Influence

**Figure 15:**
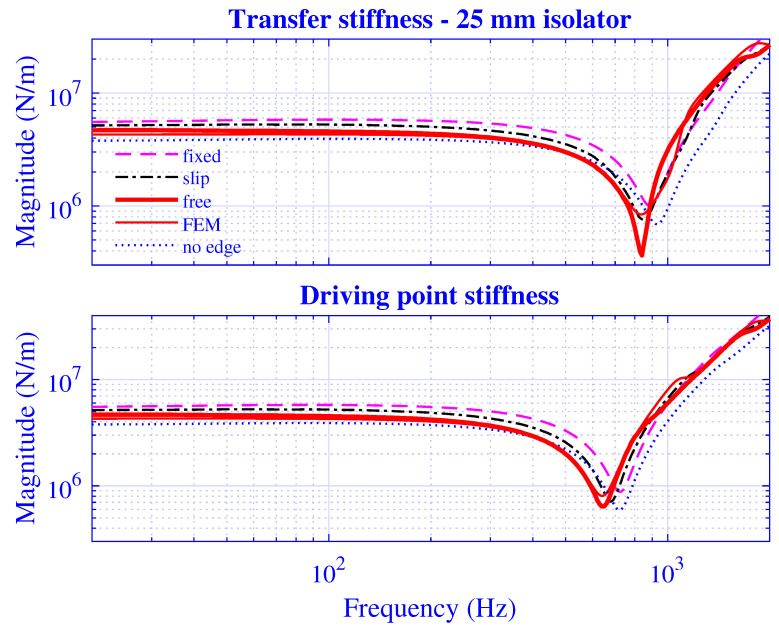
Transfer and driving point stiffness magnitude versus frequency for H=25 mm natural rubber isolator at maximum pre-compression $u_{0}=5\;$ mm while applying different boundary conditions at the edges $\partial_{t}^{1}S$ and $\partial_{t}^{2}S$ for the pre-compressed natural rubber isolator in Figure 2.

**Figure 16:**
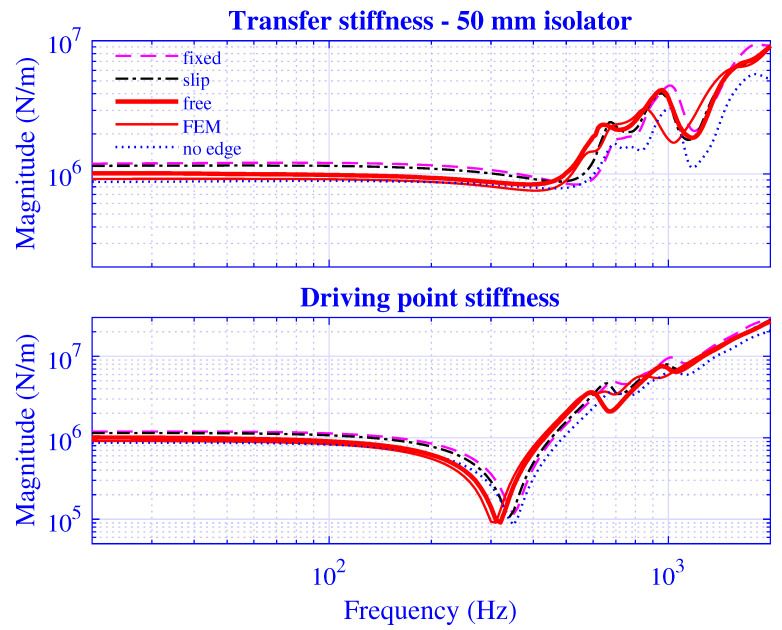
Transfer and driving point stiffness magnitude versus frequency for H=50 mm natural rubber isolator at maximum pre-compression $u_{0}=10\;$ mm while applying different boundary conditions at the edges $\partial_{t}^{1}S$ and $\partial_{t}^{2}S$ for the pre-compressed natural rubber isolator in Figure 2.

The applied boundary condition on the edges $\partial_{t}^{1}S$ and $\partial_{t}^{2}S$ for the pre-compressed natural rubber isolator in Figure 2 are *free*, that is, vanishing shear and axial stress. However, other boundary conditions may be imposed at the edges: *fixed*, that is, imposing an axial infinitesimal displacement of $-\Delta \tilde{u}e_{z}$ and vanishing radial displacement on $\partial_{t}^{1}S$ and vanishing axial and radial displacement on $\partial_{t}^{2}S$—physically representing an extension of the constraining metal plates over the whole end surfaces of the rubber cylinder in Figure 2; *slip*, that is, imposing an axial infinitesimal displacement of $-\Delta \tilde{u}e_{z}$ and vanishing shear stress on $\partial_{t}^{1}S$ and vanishing axial displacement and shear stress on $\partial_{t}^{2}S$; and *no-edge*, that is, assuming a preserved radius of the natural rubber isolator during pre-compression $u_{0}$. Although the latter condition is simple to apply, it is not physically attractive as the incompressibility condition is not fulfilled. The transfer and driving point stiffness magnitude for H=25 mm natural rubber isolator at maximum pre-compression $u_{0}=5\;$ mm are presented in Figure 15 while using the different boundary conditions—fixed, slip, free, FEM, and no-edge, where FEM represents the non-linear finite element solution from Section 3.3 of the original, pre-compressed natural rubber isolator—from Figure 1. The corresponding stiffness results for H=50 mm natural rubber isolator are presented in Figure 16 at maximum pre-compression $u_{0}=10\;$ mm. The number of modes applied is N=128 while using a threefold over-determined equation system with P=256.

Clearly, the waveguide model applying the free edge boundary condition predicts a stiffness close to that of the non-linear finite element model in Figure 15 and Figure 16. Indeed, its solution is very close to that of the finite element model almost throughout the whole frequency region—deviating only slightly within a small frequency range—whereas the fixed and slip boundary conditions overestimate the low-frequency stiffness magnitude while the no-edge condition underestimates the same. Not surprising, as a fixed and slip boundary condition should—while constraining the motion more than the free boundary condition—result in a statically stiffer natural rubber isolator whereas the thinner, no-edge natural rubber isolator results in a softer natural rubber isolator. Furthermore, the no-edge solution underestimates the stiffness magnitude in the high-frequency end, as is clearly shown in Figure 15 and Figure 16.

In summary, the developed waveguide model presented in this paper, applying a free(-free) edge boundary condition on $\partial_{t}^{1}S$ and $\partial_{t}^{2}S$, for the pre-compressed natural rubber isolator in Figure 2, is hereby in practice shown to result in a superior stiffness estimate.

### 3.6. Displacement and Stress Fields

**Figure 17:**
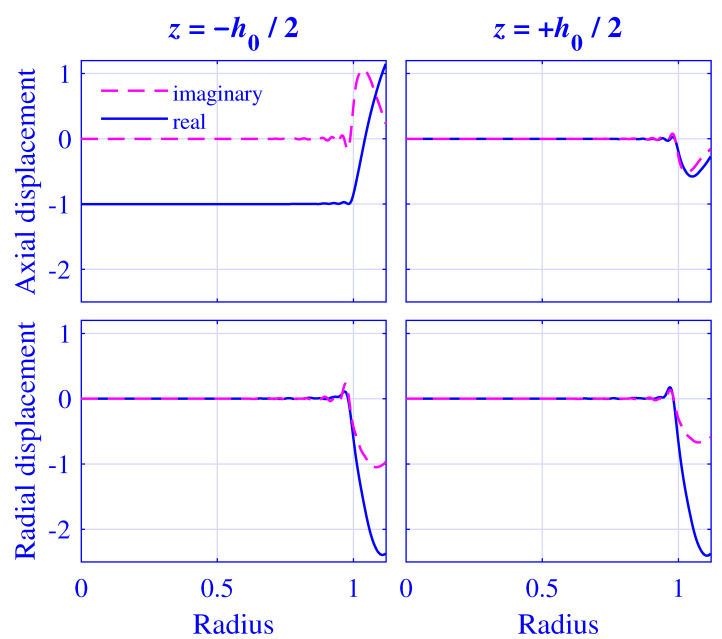
Normalized axial displacement ${\tilde{u}}_{z}/\Delta \tilde{u}$ and radial displacement ${\tilde{u}}_{r}/\Delta \tilde{u}$ at $z=\mp h_{0}\left(u_{0}\right)/2$ for H=25 mm natural rubber isolator versus normalized radius r/R, at maximum pre-compression $u_{0}=5\;$ mm and 2000Hz.

**Figure 18:**
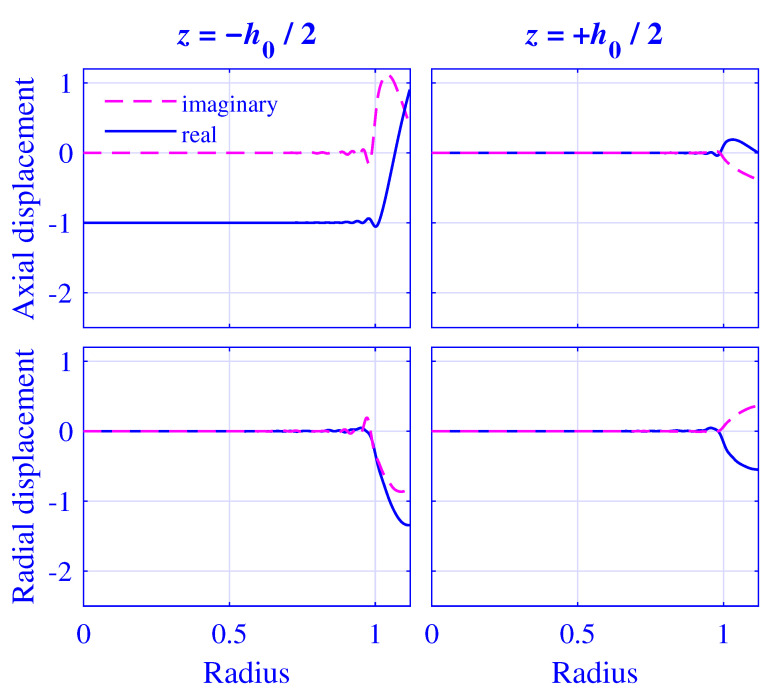
Normalized axial displacement ${\tilde{u}}_{z}/\Delta \tilde{u}$ and radial displacement ${\tilde{u}}_{r}/\Delta \tilde{u}$ at $z=\mp h_{0}\left(u_{0}\right)/2$ for H=50 mm natural rubber isolator versus normalized radius r/R, at maximum pre-compression $u_{0}=10\;$ mm and 2000Hz.

The displacement and stress fields at the cylinder ends and 2000Hz are calculated by using N=128 modes and a threefold over-determined equation system with P=256. The normalized axial and radial displacement ${\tilde{u}}_{z}/\Delta \tilde{u}$ and ${\tilde{u}}_{r}/\Delta \tilde{u}$, respectively, at $z=\mp h_{0}\left(u_{0}\right)/2$ for H=25 mm natural rubber isolator versus normalized radius r/R, at maximum pre-compression $u_{0}=5\;$ mm, are presented in Figure 17. The corresponding fields for H=50 mm natural rubber isolator, at maximum pre-compression $u_{0}=10\;$ mm, are presented in Figure 18.

Clearly, the displacement boundary conditions are well satisfied for 0≤r/R≤1 in Figure 17 and Figure 18, displaying only a slight oscillation close to r/R≈1.

**Figure 19:**
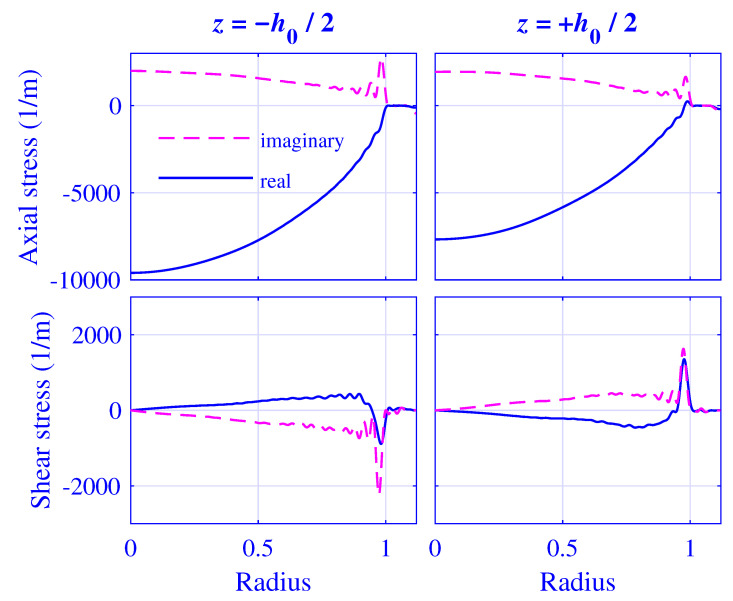
Normalized axial stress ${\tilde{\sigma }}_{zz}/\mu_{\infty }/\Delta \tilde{u}$ and shear stress ${\tilde{\sigma }}_{zr}/\mu_{\infty }/\Delta \tilde{u}$ at $z=\mp h_{0}\left(u_{0}\right)/2$ for H=25 mm natural rubber isolator versus normalized radius r/R, at maximum pre-compression $u_{0}=5\;$ mm and 2000Hz.

**Figure 20:**
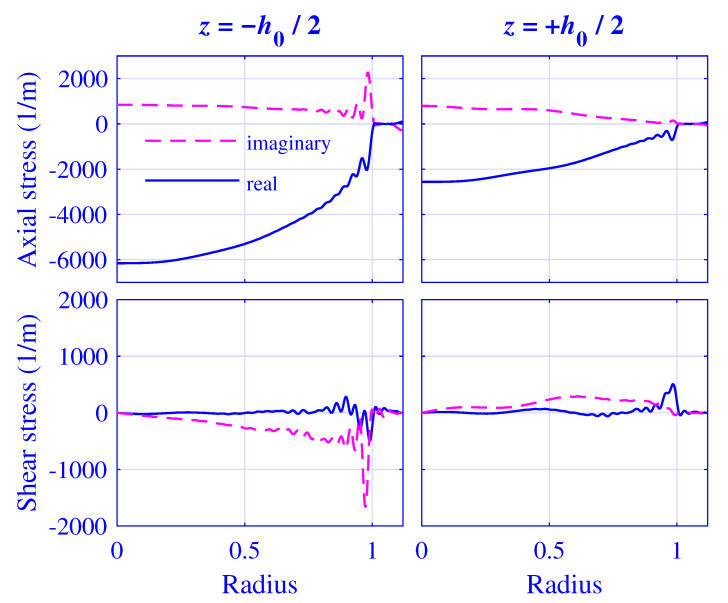
Normalized axial stress ${\tilde{\sigma }}_{zz}/\mu_{\infty }/\Delta \tilde{u}$ and shear stress ${\tilde{\sigma }}_{zr}/\mu_{\infty }/\Delta \tilde{u}$ at $z=\mp h_{0}\left(u_{0}\right)/2$ for H=50 mm natural rubber isolator versus normalized radius r/R, at maximum pre-compression $u_{0}=10\;$ mm and 2000Hz.

Likewise, the normalized axial and shear stress ${\tilde{\sigma }}_{zz}/\mu_{\infty }/\Delta \tilde{u}$ and ${\tilde{\sigma }}_{zr}/\mu_{\infty }/\Delta \tilde{u}$, respectively, at $z=\mp h_{0}\left(u_{0}\right)/2$ for H=25 mm natural rubber isolator versus normalized radius r/R, at maximum pre-compression $u_{0}=5\;$ mm, are presented in Figure 19. The corresponding fields for H=50 mm natural rubber isolator, at maximum pre-compression $u_{0}=10\;$ mm, are presented in Figure 20.

Clearly, the stress boundary conditions are well satisfied for $1<r/R\leq r_{0}\left(u_{0}\right)/R$ in Figure 19 and Figure 20. In addition, the shear stress ${\tilde{\sigma }}_{zr}={\tilde{\sigma }}_{rz}$ vanishes identically at $r/R=r_{0}\left(u_{0}\right)/R$ due to the stress boundary condition applied at the radial surface while deriving the dispersion relation (25). However, the axial stress is singular $|{\tilde{\sigma }}_{zz}|\to \infty$ at r/R=1. This point value is impossible to satisfy using the proposed method while using non-singular terms unless N→∞. However, the axial force needed for the dynamic stiffness evaluation is determined by a spatial integration of the axial stress (see Equations (63) and (64)) which is not that sensitive to singular points, as is clearly shown in Section 3.3 and Section 3.4.

In addition to the unfilled natural rubber material studied in this paper, the developed model is most likely suitable for other incompressible and nearly incompressible polymer materials where, moreover, the Mittag-Leffler stress relaxation function is suitable, such as for styrene-butadiene rubber (SBR) [45] and tough, doubly cross-linked, single network hydrogels with both chemical and physical cross-links [64] and where, furthermore, the geometrical non-linearity prevails over the material non-linearity, such as for not overly filled polymers.

## 4. Conclusions

A waveguide model is developed for the dynamic stiffness of pre-compressed natural rubber vibration isolators by transforming the isolator into a globally equivalent homogeneous and isotropic form, where the boundary conditions are satisfied by mode matching. The conclusions are as follows:

- The dynamic stiffness solutions are shown to converge for a moderately high number of modes—such as N≥64 for the studied vibration isolators within the considered frequency range 20 to 2000 Hz and pre-compression range up to 20%;
- The dynamic stiffness is found to depend strongly on the frequency displaying resonance phenomena such as peaks and troughs;
- The dynamic stiffness is found to depend strongly on the pre-compression, displaying low-frequency stiffness magnitude stiffness increase in addition to peak and trough shifts with increased pre-compressions;
- The waveguide model is shown to yield dynamic stiffness results close to those of the non-linear finite element method;
- The non-linear finite element method was previously shown to give results close to those of the experiments for similar natural rubber vibration isolators [11];
- The applied Mittag–Leffler shear relaxation function was previously shown to give shear modulus results close to those of the experiments for the same natural rubber material as studied in this paper [20]; and
- The free-free cylinder edge boundary condition is shown to result in superior pre-compressed dynamic stiffness results.

An interesting continuation of the work presented is to apply the proposed method to other excitation directions, such as torsional and shear, and to other natural rubber vibration isolators, such as strips and rectangular shaped natural rubber vibration isolators. Another possible extension is to additionally include physically and chemically aging of the natural rubber, for example, by applying shear modulus models that incorporate physical and chemical aging [52,53].

## Figures and Tables

**Table 1:** Ingredients of the natural rubber material and their concentrations [20].

Ingredient	Kind	Concentration [phr]
Natural rubber	GP SMR	100
Stabilizers	Wax	1
	Antioxidant	1
	Antiozonant	1
Activators	Zinc oxide	5
	Stearic acid	1
Vulcanizing agent	Sulphur	3
Accelerator	CBS ${}^{1}$	2
Processing oils	Aromatic	5
	Paraffinic	1

^1^*n*-Cyclohexyl-2-Benzothiazole Sulfenamide.

**Table 2:** Applied natural rubber material parameters in the model [20].

Quantity	Variable	Value
Density	ρ	984 kg/m${}^{3}$
Nearly incompressible parameter	α	2222
Equilibrium shear modulus	$\mu_{\infty }$	825 kN/m${}^{2}$
Relaxation density	Δ	276
Generalized relaxation time	τ	2.94 ns
Fractional derivative order	β	0.657

## Data Availability

All data are included within the text.
